# The Colorado LEAP study: rationale and design of a study to assess the short term longitudinal effectiveness of a preschool nutrition and physical activity program

**DOI:** 10.1186/1471-2458-13-1146

**Published:** 2013-12-09

**Authors:** Laura L Bellows, Susan L Johnson, Patricia L Davies, Jennifer Anderson, William J Gavin, Richard E Boles

**Affiliations:** 1 Colorado State University, Department of Food Science & Human Nutrition, 1571 Campus Delivery, Fort Collins, CO 80523-1571, USA; 2 Department of Pediatrics, Section of Nutrition, University of Colorado Denver Anschutz Medical Campus, Aurora, CO 80045, USA; 3 Department of Occupational Therapy, Colorado State University, Fort Collins, CO 80523, USA; 4 Department of Human Development & Family Studies, Colorado State University, Fort Collins, CO 80523, USA

**Keywords:** Obesity, Social ecological model, Preschool, Early childhood, Intervention, Longitudinal

## Abstract

**Background:**

The preschool years are a critical window for obesity prevention efforts; representing a time when children establish healthy eating habits and physical activity patterns. Understanding the context in which these behaviors develop is critical to formulating a model to address childhood obesity. The Colorado LEAP Study, an intervention study designed to prevent early childhood obesity, utilizes a social ecological approach to explore individual, family and environmental factors and their relationship to child weight status over a 3 year timeframe.

**Methods:**

The study is located in 5 rural Colorado preschool centers and elementary schools (2 treatment and 3 control). Treatment sites receive *The Food Friends®* nutrition (12 weeks) and physical activity (18 weeks) interventions during preschool. Observational measures assess 3 layers of the social ecological model including individual, family and organizational inputs. Children’s food preferences, food intake, gross motor skills, physical activity (pedometers/accelerometers), cognitive, physical and social self-competence and height/weight are collected. Parents provide information on feeding and activity practices, child’s diet, oral sensory characteristics, food neophobia, home food and activity environment, height/weight and physical activity (pedometers). School personnel complete a school environment and policy assessment. Measurements are conducted with 3 cohorts at 4 time points – baseline, post-intervention, 1- and 2-year follow-up.

**Discussion:**

The design of this study allows for longitudinal exploration of relationships among eating habits, physical activity patterns, and weight status within and across spheres of the social ecological model. These methods advance traditional study designs by allowing not only for interaction among spheres but predictively across time. Further, the recruitment strategy includes both boys and girls from ethnic minority populations in rural areas and will provide insights into obesity prevention effects on these at risk populations.

**Trial registration:**

ClinicalTrials.gov: NCT01937481.

## Background

Childhood obesity continues to be a major public health concern, with rates increasing over the last thirty years [1]. Currently, more than 26% of preschool-aged children are considered either overweight or obese. Children and families from low socioeconomic and minority backgrounds; particularly in rural areas that have limited access to food, activity & health-related services, are disproportionately affected [1,2]. Despite the high need, early childhood has not been a focus of obesity prevention efforts [3].

The preschool years present a critical window in which to begin obesity prevention efforts as they represent a time when young children establish healthy eating habits and physical activity patterns [4]. Unfortunately, young children are not meeting nutritional or physical activity guidelines [5,6]. Because early childhood is one of rapid development, it may afford the best opportunities for altering development in ways that can reduce obesity risk [3]. Food preferences are developed *in utero* and in early childhood and tend to persist throughout life [7,8]. Cross-sectional research has demonstrated that children who are more willing to try novel foods and who are less food neophobic have higher quality diets than those children who are more food neophobic [9].

The causal factors contributing to low activity levels among preschoolers are not well understood, however it is posited that there may be a relationship between the status of children’s motor skill performance and their levels of physical activity [10,11]. Several studies have reported that children with poorer motor skill performance were less active than children with better-developed motor skills [10-14]. Interventions aimed at altering developmental pathways, such as influencing food neophobia and gross motor proficiency, may impact dietary consumption and physical activity levels in later childhood and have long term impacts on the risk for obesity. The most informative designs regarding the development of behaviors associated with eating and activity outcomes necessitate a longitudinal design which samples a variety of behavioral antecedents and outcomes.

*The Food Friends* program is one such intervention that has addressed developmental behaviors in young children. Comprised of two components *– Fun with New Foods®* and *Get Movin’ with Mighty Moves®* - the program has successfully demonstrated increases in both children’s willingness to try new foods (food preference) and gross motor performance in preschool-aged children [15,16]. Further exploration is warranted to understand social and environmental influences on these behaviors.

Children’s eating and activity behaviors are influenced by factors that are individual to the child, but also by the environments, in which they live, learn and play. Ecological models provide a framework for addressing interactions across individual, social and environmental spheres of influence [17,18]. Predictive behaviors within these environments can influence child behaviors. While many predictive behaviors have been shown to influence dietary intake, physical activity, and weight status; food preference and motor performance merit further exploration. Understanding the context in which child behavioral patterns are developed is critical to developing a model to address childhood obesity. Skouteris [19] makes the argument that children’s development is a bidirectional transaction, with parents influencing children but also children impacting parents’ decisions and behaviors. Understanding environments, parent behaviors and children’s behaviors over time will provide an opportunity to begin to develop transactional models.

The Colorado Longitudinal Eating And Physical activity (LEAP) Study utilizes a social ecological approach to explore individual, family and environmental factors and their relationship to child weight status over a 3 year timeframe. Our primary research questions are as follows:

1. Are behavior changes (e.g. increased willingness to try new foods and improved gross motor skills) positively influenced by a preschool nutrition and activity program, *The Food Friends®*, sustained through early elementary school?

2. Do *The Food Friends®* programs have an impact on reducing the percentage of children considered overweight and/or obese over a 3 year timeframe?

3. Do food preference and gross motor performance directly affect child weight status or are they mediators to dietary intake and physical activity?

## Methods/design

### Study design

**Figure 1:**
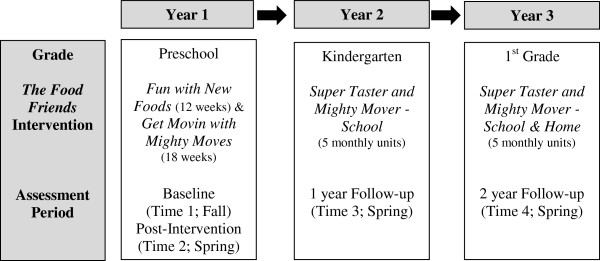
Colorado Longitudinal Eating And Physical activity (LEAP) study design.

The Colorado LEAP project is a longitudinal cohort study utilizing a controlled quasi-experimental design in 5 rural Colorado communities. Two communities serve as intervention communities with the other 3 as matched controls. Intervention sites will receive *The Food Friends®* nutrition and physical activity programs in preschool and ‘booster’ programming in kindergarten and 1^st^ grade. Assessments will be administered 4 times - twice in preschool (Fall and Spring) and once in both kindergarten and 1^st^ grade (Spring) (Figure 1). Observational measures of children will be conducted at the school; parent/home measures will be sent home and returned to school via the child; and school personnel will complete school environment and policy assessments. This study was approved by the institutional review boards at Colorado State University and the University of Colorado Denver Anschutz Medical Campus.

### Participant recruitment

Families will be recruited via an informational and consent packet sent home with their 4-year-old child and during Parent Information events to be held at the preschools. The packet will be available in Spanish and in English and both English- and Spanish- speaking staff will be present during recruitment events to be inclusive of all families whose children attend the preschool. Written consent will be obtained from parents or guardians prior to enrollment in the study. Children with identified developmental issues (e.g. cerebral palsy and downs syndrome) will be included in study activities but removed from data analysis.

### Sample size estimation

This study is powered on mean change differences in gross motor skills between intervention and control children. The statistical evaluation associated with the research questions will employ either Multivariate Analysis of Variance (MANOVA) or Analysis of Variance (ANOVA) procedures followed by planned comparison t-tests. As such, a power analysis based on the formulas and tables proposed by Cohen [20] shows that to evaluate motor performance in children using the results of ANOVAs on gross motor measures reported by Bellows et al. [16], a sample size of 90 per group could detect significant differences in motor performance between the two groups with 90% power. A power analysis for changes in BMI with an improvement of .5 standard deviation by the intervention group over the control group (ES = .50) and a power level set at .80 would need a sample size of 64 per group to show statistical significance at α = .05. Further, because differences in assessing changes in food exposure have been detected in samples as low as n = 12, the power for this study was based upon motor skills. Thus, with 90 children per group the power is high enough to detect changes in motor performance while also having a sensitive environment to pick up effects on the nutrition measures. Finally, to account for potential 10% attrition rate, as seen in our previous studies, we are planning a potential study size of 200 children; that is, 100 children in each of the two convenience samples.

### Intervention

*The Food Friends®* is a research-based preschool program designed to address childhood obesity by establishing healthful eating and physical activity behaviors in preschool-aged children. The program consists of two components: *The Food Friends: Fun With New Foods®* focuses on helping children increasing children’s willingness to try new foods and *The Food Friends: Get Movin’ With Mighty Moves®* aims to enhance preschoolers’ gross motor skill development. The program utilizes constructs of the social cognitive theory, tenets of social marketing, and is embedded within Bronfenbrenner’s social ecological framework [21]. Two study schools will receive the intervention in preschool and elementary school (kindergarten and 1^st^ grade) while the three schools in the control condition will receive standard curriculum.

#### Preschool: The Food Friends ®

*The Food Friends: Fun with New Foods (FWNF)* classroom program is a 12-week intervention developed by Colorado State University [22-26]. The length of the program is critical and was established from literature reviews and our own project evaluation data, confirming that 8–12 experiences are necessary for a child to try and accept a new food [15]. Classroom implementation will include: a 15–20 min nutrition activity 1×/week and opportunities to try new foods 2×/week. Various tactics bring *The Food Friends* to life in a playful and exciting manner. Child-centered activities and supporting materials were developed for 8 food characters and 13 novel foods [22,26]. Eight *Food Friends* characters are central to program themes and materials.

*The Food Friends: Get Movin’ with Mighty Moves (MM)* Classroom Program is 18 weeks in length, and will be conducted 4 days a week for 15–20 min/day, for a total of 72 lessons led by the teacher [27]. Each week will focus on a skill from 1 of the 3 gross motor skill categories: stability (e.g., balance), locomotor (e.g., running and jumping), or ball skills (e.g., throwing and kicking). Early in each week, children will be introduced to a motor skill with movement concepts added as the week progresses. Later in the program, skill patterns, a combination of more than one motor skill, will be incorporated into activities. Creatively, graphics depicting each of *The Food Friends* characters participating in different physical activities are incorporated throughout program materials. They illustrate the different motor skills presented to children as *Mighty Moves*. Both programs will utilize a superhero theme (Super Taster and Mighty Mover) to engage in desired behaviors in a fun and creative manner [27,28].

Teacher training at intervention schools will be conducted for both programs prior to classroom implementation addressing program materials, concepts, and child feeding and activity topics. Feeding topics will include neophobia, eating characteristics of 3- to 5-year olds, role modeling, and how to encourage children to try new foods. Hands-on and participatory activities demonstrate to teachers various ways of teaching motor skills and the *Mighty Moves* activities.

Bilingual home connection materials encourage parents, through a set of Simple Tips, to create an environment that provides children with multiple opportunities to learn about and try new foods at home and to participate with their children in physical activity [24,29]. These tips will be seen repeatedly through child-centered parent materials, including: handouts, placemats, an activity book, trading cards, the *Mighty Moves* musical CD with poster insert.

#### Elementary school: Super Tasters and Mighty Movers

In effort to sustain the preschool behavior changes, the messages from *The Food Friends®* (Super Taster and Mighty Mover) will be extended into early elementary school through a ‘booster’ program. The booster program will consist of a kindergarten and 1^st^ grade curriculum with 5 monthly units. Units will include short reminder activities and lessons that reinforce the program messages learned in the preschool *FWNF* and *MM* programs. Banners and posters will be displayed throughout the school environment, including classrooms, cafeteria, and the gymnasium.

To promote school-based messages in the home environment, five monthly packets of The Super Taster and Mighty Mover Club, will be mailed to 1st grade study participants. Each of the mailings will consist of a child newsletter, a bilingual parent newsletter, and a branded educational enhancer (e.g. jump rope, spatula, etc.).

### Measurements

**Figure 2:**
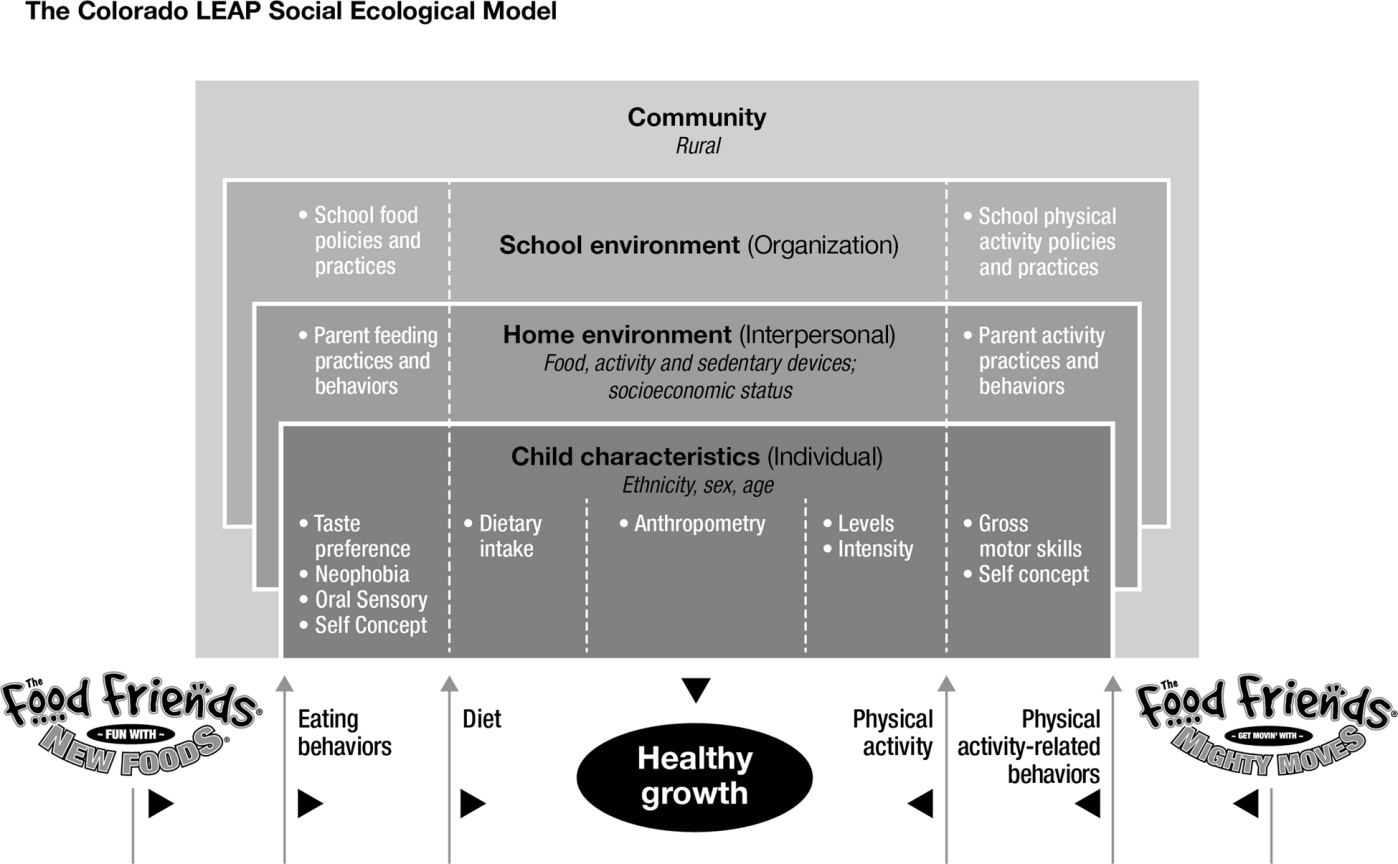
**Conceptual model for the Colorado LEAP study.** This model illustrates a multidimensional approach to addressing healthy growth in young children. The bottom of the figure depicts the intervention’s impact on Eating and Physical activity-related behaviors which then may influence Diet, Physical Activity, and Growth. These behaviors occur in multiple environments (micro-, meso-, & macrosystems; colored boxes) that are influenced by roles and social norms. Individual and environmental characteristics (black font) may interact with behaviors, practices, and/or policies (white font) to influence healthy growth. The items listed in the white font in the Child characteristics and Home environment boxes are the outcome measures used in this study.

Assessments for the LEAP study are within and across 3 spheres of the social ecological model (Figure 2). Assessments sent home to parents are available in either English or Spanish.

### Eating behaviors

#### Taste preference (Willingness to try new foods)

Children’s willingness to try new foods and liking for foods will be assessed via a preference assessment administered to each child [30]. The child will be asked to taste 9 foods in a self-selected order, 5 thought to be familiar foods and 4 thought to be novel foods for most young children. Foods will include jicama, garbanzo beans, grapefruit, Gouda cheese, couscous, spinach, salmon, beets, and pineapple; thus representing sweet and savory foods, fruits, vegetables, protein, foods introduced by *The Food Friends*, known and new foods. Children will rate the food as: “yummy”, “just ok” “yucky” using faces that display these qualities. Food refusals will be recorded, obtaining willingness to try (refusal rate) and a liking rating for each food [30].

#### Food neophobia

The Reaction to Food scale of the Colorado Childhood Temperament Inventory provides caregivers ratings of their child’s neophobia and child temperament related to food and eating [31]. This scale consists of 5 items and assesses children’s food dislikes, including new foods (responses range from 1=“not at all like my child” to 5=“a lot like my child;” range of scores from 5 – 25). The measure is reported to have good internal consistency (α = 0.77), test-retest reliability (r = 0.74) and concurrent validity with other longer measures of child neophobia (r = 0.83 with the Food Neophobia Scale (FNS)) [32]. The FNS (child version) [33], an additional parent assessment of the trait of child neophobia, will be administered to parents. The FNS is a 10 item test that assesses children’s willingness to eat foods (responses range from 1=“Agree Strongly” to 7=“Disagree Strongly” with a range of scores from 10–70). This measure has been validated against behavioral observations of children’s willingness to taste foods (r = 0.38, p ≪ 0.001) and parent predictions of their child’s willingness to try foods (r = 0.34, p ≪ 0.001).

#### Oral sensory characteristics

The Sensory Profile is a 125-item questionnaire which measures sensory processing in 6 different domains, including oral sensory sensitivity [34]. The twelve items that are part of the Oral Sensory Processing scale (e.g. “Avoids tastes or food smells that are typically part of a children’s diet”; internal consistency, α = 0.845) will be utilized for this study. Responses for the Sensory Profile are given on a 5-point Likert scale (Always = 1 to Never = 5) and it has been used and validated with both clinical and non-clinical samples of children [35-37]. Scores for the oral sensory processing scale used in this study can range from 12 to 60 and higher scores relate to typical performance in oral sensory processing and lower scores indicate that there are issues in oral processing.

#### Self concept

The Pictorial Scale of Perceived Competence and Social Acceptance for Young Children (PSPCSA) is a self-report instrument designed to assess perceptions of young children ages four to seven in four domains (i.e., Cognitive Competence, Physical Competence*,* Peer Acceptance, Maternal Acceptance) [38]. The test uses a structured response format that is designed to be sensitive to the developmental capacities of young children, allowing children to identify with test items, and to make meaningful differentiations between possible responses. Each of the four subscales contains six test items for a total test length of 24 items. Oblique factor analyses indicated that intercorrelations among the items for preschool and K-2 children provide evidence of the factorial validity of the instrument. Reliability coefficients fell between .52 and .85. Harter and Pike provided evidence that the PSPCSA showed good convergent, discriminant, and predictive validity in preschool and K-2 children [39].

#### Parent feeding practices and behaviors

Child Feeding Questionnaire v.2 (CFQ) is a self-report measure that assesses child-feeding attitudes and practices and perceptions of the child’s weight. Parent feeding practices are measured using 5 subscales from the original CFQ (Restriction, Pressure to Eat, Monitoring, Concern about Child Weight) and 3 additional scales that have been developed in samples in Colorado (Offering New Foods, Urging New Foods, Concern about Child not Eating Enough). Items are scored on a Likert scale of 1 = Never to 5 = Always. The CFQ has demonstrated good internal consistency, test retest reliability and convergent validity [40].

### Diet

#### Consumption of program target foods

In preschool classrooms, pre-weighed servings (65 ± 2 g) of 2 foods - a program indicator food (jicama) and a novel food (edamame) – will be offered at lunchtime (preschool) or snack time (elementary school) along with other lunch or snack foods. Each child’s target foods will be post-weighed and the amount consumed recorded in g.

#### Food frequency

The Block Kids Food Screener (Ages 2 – 17) will be used to measure parent reports of their child’s food intake. This food screener assesses children’s intake by food group and reports outcomes in number of servings and calories consumed [41,42].

### Healthy growth

#### Anthropometrics

Children’s weight and height will be measured according the method of Harrison and colleagues [43] on a digital scale (Lifesource ProFit UC321; Milpitas, CA) to the nearest 0.05 kg (.1 pounds) and by portable stadiometer to the nearest 0.1 cm (Seca Corp, Hamburg, Germany) by trained research staff. Body Mass Index (BMI) and sex- and age-adjusted BMIz scores will be calculated in the manner documented in the 2000 CDC Growth Charts for the United States [44]. Parental height and weight is collected by self-report, and BMI is calculated.

### Physical activity

#### Physical activity levels and intensity

Pedometers and accelerometers will used to assess physical activity (PA). Two Walk-4-Life pedometer (Model W4L Classic; Plainfield, IL), with safety strap, will be sent home along with a log to record the number of daily steps. Parents will be asked to place the pedometer on the child and themselves when the child/parent gets out of bed and to take it off when the child/parent goes to bed. They will be asked to record the number of total steps taken each day for six days – four weekday and two weekend days. Parents will also be asked to record notes about if the child/parent did not wear the pedometer for a specific time during the day, what types of activities they participated in, etc. A subsample of participants will be asked to wear an Actical accelerometer (Phillips – Respironics, Oregon, USA) to measure time spent in various intensities of PA as well as time spent in activity at school versus home. The accelerometer will be attached on the non-dominant wrist of each student with semi-non-removable band to measure PA. The Actical is a small, lightweight, waterproof, omni-directional accelerometer. It will be placed on the child during the school day. Children will be asked to wear the accelerometer for a 6-day period and to maintain “typical” activity patterns. To encourage compliance in wearing the accelerometers, the children and parents will be given written and verbal instructions.

### Physical activity related behaviors

#### Gross motor skills

The Bruininks-Oseretsky Test (BOT-2) of Motor Proficiency, Second Edition, will be used to assess gross motor development [45]. The BOT-2 is a standardized, norm-referenced measure for children ages 4 through 21 years of age. Reliability and validity of the BOT-2 is reported in the test manual [46]. Four subtests will be performed with children: balance; speed and agility; upper limb coordination (ball skills); and strength. Administration and scoring will be conducted according to the standardized methods described in the manual. Testing time averages between 20–25 min. Raw scores for each sub-test will be calculated and then transformed into standard scores.

#### Parent activity practices and behaviors

This physical activity (PA) survey will be completed by parents. It addresses the parent’s perception of the child’s athletic coordination and enjoyment of PA. The survey also will inquire about parent enjoyment of PA, family support for PA, and importance of the child participating in sports and PA and parent PA, including moderate PA during the previous 7 days, vigorous PA during the previous 7 days, and a self-rating (0 to 10) of parent’s PA level [47]. The survey is a hybrid of the valid and reliable Amherst Health and Activity Study Adult Survey of Child Health Habits used in 5–12 y olds and an adaption of the Amherst Survey used in 3- to 5-y olds [48,49].

### Home and school environments

#### Home food and activity environment

The Home Inventory Describing Eating and Activity (Home IDEA) is a self-report parent survey of the physical home environment. This measure has been modified from an existing measure with established reliability (inter-rater) and validity (criterion and construct) [50-52]. The Home IDEA has been adapted to include a greater variety of foods and drinks, including representation of items for families having low socio-economic resources and living in rural environments. The Home IDEA includes responses for the presence of food and drinks (108 items), PA devices (16 items), and measures the child’s bedroom for the presence of electronic devices (e.g. television; 12 items).

#### Preschool environment

Key preschool staff will complete the Nutrition and Physical Activity Self-Assessment for Child Care (NAP SACC) instrument to identify current center nutrition and physical activity policies and practices. The self-assessment instrument includes 54 items covering nine nutrition and five physical activity areas. Nutrition areas include 37 questions covering such topics as foods offered, menus and variety, feeding practices, education for staff, children and parents; and nutrition policy. Physical activity areas include 17 questions addressing: active play and inactive time; play environment; supporting physical activity; physical activity education for children, parents, and staff; and physical activity policy. The NAP SACC instrument has been used in previous studies measuring nutrition and physical activity policies practices in early childhood settings and has documented evidence of validity and reliability [53-56].

#### School environment

The School Environment and Policy Survey, consists of three modules designed to capture nutrition and physical activity environments, policies and programs offered in the elementary schools. It will be completed by the Principal, Food Service Manager and Physical Education Teacher at each participating school. This instrument covers the following content areas: opportunities for healthy eating in the cafeteria; opportunities for healthy eating outside of the cafeteria; healthy eating policies and practices; increasing physical activity through physical education; increasing physical activity through recess; other opportunities for physical activity; and other school policies and practices [57].

### Demographic information

Parents will also complete an additional questionnaire that supplies demographic information including age, ethnicity, race, education, employment status, and income bracket.

### Data collection

Observational measures of children conducted at the schools and parental responses to the various assessment instruments returned in the parent packet will be aggregated into a single master database using a variety of strategies. While some assessment measures will be collected using traditional paper forms, two other methods have been developed to increase the accuracy while reducing time for data entry into the database.

First, electronic forms designed and implemented in Microsoft Access program running on 8 touch-tablet PCs are used to record child performance or answers during assessments conducted in the school settings. This approach uses a split-database design in which data-entry forms are dispersed as file copies residing on each touch-table PC and all data, organized in several tables, reside in a single back-end Access database file. To upload the data collected on the customized electronic forms, the touch-tablet PCs are networked via Wi-Fi to a notebook computer functioning as an ad hoc router/server where the back-end database file resides. This split-database design eliminates the problem of how to collate the data off each tablet into a unified database.

A second alternative method for electronic data entry was developed for the parent reports when the nature of the responses could be organized to be answered as checked off items. For this approach, we specifically designed the assessments instrument using Microsoft Word software to be electronically scanned using the Remark Office OMR 8 Software (Gravic, Inc., Malvern, PA). Scanned records were then added to the master database.

Finally, to reduce data entry errors all measures recorded on paper forms that have to be manually entered into the database, we designed electronic entry forms in Access to match the appearance of the original paper form. To further ensure the accuracy of entries, measures will be independently entered into a second database file and each measure entered into the master database will be compared to their corresponding second entry. Any discrepancies found will be resolved by referring to the original paper form with the correct values re-entered.

### Data analysis

Data will be analyzed using SPSS 21.0 Windows (IBM SPSS Statistics, Inc., Chicago, IL) program. Descriptive statistics (e.g., percentage, mean, standard error, etc.) will be obtained for demographic, anthropometric, and weight status variables. Additionally, shape-based statistics will be calculated to test assumptions for parametric procedures; data not meeting assumptions will either be transformed or appropriate non-parametric procedures will be utilized. The statistical evaluation associated with the research questions will employ either MANOVA or ANOVA procedures followed by planned comparison t-tests. For example, to answer Question 1 that states *Food Friends®* and *Mighty Moves*™ increase willingness to try new foods and develop motor performance, and that these gains will persist throughout a three year period, a 2x2 MANOVA mixed model design will be used to assess the final outcome measures at the end of the last year. The two factors, are Treatment Intensity (2 levels; experimental and control groups), a between group factor, and Time (2 levels; pre and post treatment), a within group factor. The multiple dependent measures to be explored include the nutrition measures of food preferences, willingness to try, and dietary consumption along with physical activity measures including for example, gross motor skills, pedometer steps and accelerometer outputs. To test the specific hypotheses for Question 1, each dependent variable will be explored using 2x4 ANOVA design (the same factors as above but the within factor, Time, consisting of 4 time periods) followed by orthogonal *a priori* t-tests. Covariates will also be added to the basic ANOVA design to explore effects of age, sex, ethnicity, and location. The significance level will be set at α = 0.05 for all analyses.

To assess whether preference and motor performance directly affect child weight status or serve as mediators to dietary intake and physical activity as they affect child weight (Question 3), three prediction models will be tested beginning with the use of a hierarchical multiple regression analysis. In both prediction models, measures representing concepts of Davison and Birch’s ecological model of child weight status serve as predictors (i.e. independent variables) with measures of child characteristics and child risk factors entered first (step 1), and measures of parenting styles and family characteristics in the next step, (step 2). The prediction models will differ in their inclusion or exclusion in step 1of (a) dietary intake and physical activity measures or (b) taste preference and motor performance or (c) the combination of dietary intake, physical activity measures, taste preference and motor performance. Changes in the beta weights of these variables across the 3 predictor models will be examined to determine the relationships of taste preference to dietary intake and motor performance to physical activity measures.

## Discussion

Currently, studies are beginning to examine behaviors and correlates related to children’s weight status across multiple levels of the social ecological model [58], however most have been cross-sectional in nature [7,59]. Longitudinal studies are needed to identify causal influences of children’s dietary and physical activity behaviors as well as on growth [19]. Examining eating and activity behaviors longitudinally, and in multiple environments, can provide insights into relationships between predictive behaviors and dietary, physical activity and weight outcomes.

The Colorado LEAP study utilized Davison and Birch’s ecological model of childhood obesity as a springboard for the design of the study, which allows for longitudinal exploration of relationships among eating habits, physical activity patterns, and weight status within and across spheres of the social ecological model. The methods represent an advance in study design as they will allow not only for interaction among spheres but will do so predictively across time. In addition to the primary study outcomes examining behavior changes in children, the design allows for exploration into what parents report at baseline and its impact on child behaviors over time, and conversely child behaviors at baseline and the impact on parent response at future time points. Examining the data in this way supports Skouteris et al.’s [19] viewpoint of children’s development being bidirectional.

Key strengths of this study include utilization of a multi-disciplinary approach and a longitudinal design embedded within a social ecological framework. A further strength is that the recruitment strategy includes both boys and girls from ethnic minority populations in rural areas and will provide insights into obesity prevention effects on these at risk populations. It should be noted that while schools based in rural communities were purposefully chosen as study sites, a limitation is that we do not include a measure assessing the obeseogenic environment at the community level. Ultimately, the design of the Colorado LEAP Study has the potential to inform strategies for childhood obesity interventions in early childhood.

## Abbreviations

LEAP: Longitudinal eating and physical activity; MANOVA: Multivariate analysis of variance; ANOVA: Analysis of variance; ES: Effect size; FWNF: The Food Friends: Fun with New Foods; MM: The Food Friends: Get Movin’ with Mighty Moves; FNS: Food neophobia scale; PSPCSA: Pictorial scale of perceived competence and social acceptance for young children; CFQ: Child feeding questionnaire v.2; BMI: Body mass index; PA: Physical activity; BOT-2: Bruininks-Oseretsky test of motor proficiency, second edition; Home IDEA: Home inventory describing eating and activity; NAP SACC: Nutrition and physical activity self-assessment for child care.

## Competing interests

The authors declare that they have no competing interests.

## Authors’ contributions

LB is the PI and developed the first draft of the manuscript. SLJ, PLD, JA, WJG, and REB contributed to the conception and design of the study. SLJ, PLD, WJG, and REB contributed to drafting the manuscript and provided critical input. All authors have read and approved the final manuscript.

## Pre-publication history

The pre-publication history for this paper can be accessed here:

http://www.biomedcentral.com/1471-2458/13/1146/prepub
